# Health Literacy and Self-care among Pregnant Women in a Low Income Country: A Pilot Study in Indonesia

**DOI:** 10.4314/ejhs.v35i5.3

**Published:** 2025-09

**Authors:** Nurul Fadhilah Gani, Ilmawanti, Nurhidayah, Hasnah, Nordianna Seman

**Affiliations:** 1 Department of Nursing, Faculty of Medical And Health Sciences, Universitas Islam Negeri Alauddin Makassar, Makassar City, Indonesia; 2 Center for Nursing Studies, Faculty of Health sciences, MARA University of Technology

**Keywords:** Pregnancy, Health literacy, Self-care

## Abstract

**Background:**

Self-care during pregnancy is a crucial factor influencing the health of pregnant women. Maternal complications are often associated with low self-care behavior during pregnancy. The ability and awareness of pregnant women play a significant role in fostering good self-care, which is closely linked to health literacy. Unfortunately, in Indonesia—a low-income country—health literacy among pregnant women remains relatively low, and limited research has addressed this issue. This study aimed to determine the relationship between health literacy and self-care among pregnant women.

**Methods:**

A quantitative study with a cross-sectional design was conducted, involving a population of 80 postpartum mothers. A total of 66 participants were selected using a non-probability purposive sampling technique. Data were collected through two questionnaires: the Maternal Health Literacy and Pregnancy Outcome Questionnaire (MHLPAQ) to measure maternal health literacy, and a self-care questionnaire developed by researchers to assess self-care practices. The Spearman test was employed for data analysis. The study was carried out at the Kassi-Kassi Public Health Center from May 10 to June 7, 2024.

**Results:**

The findings revealed a significant relationship between health literacy and self-care (r = 0.849; p-value < 0.001), indicating a very strong positive correlation. This demonstrates that health literacy is closely associated with self-care among pregnant women at the Kassi-Kassi Public Health Center, Indonesia.

**Conclusion:**

Higher health literacy is associated with better self-care behavior among pregnant women. Therefore, enhancing health literacy is essential for improving maternal self-care practices in Indonesia.

## Introduction

Pregnancy is a natural process experienced by women, involving numerous physical and psychosocial changes until childbirth due to the growth and development of the fetus. These changes may result in various problems and complications; therefore, appropriate interventions are necessary. Physiological changes during pregnancy include alterations in blood pressure, increased nutritional needs, changes in the urinary system, sleep patterns, and other modifications that affect nearly all body systems (1).

Other challenges commonly experienced by pregnant women include neglecting personal hygiene, breast care, immunization, pregnancy exercises, antenatal check-ups, and adequate nutrition for fetal development. Such neglect can adversely affect fetal growth and development and may even lead to complications and mortality (2,3). Almost all changes occurring during pregnancy can be managed by improving a mother's self-care ability.

Self-care behavior contributes to reducing morbidity and mortality, improving quality of life, and lowering pregnancy-related healthcare costs. Essential self-care activities empower women to manage the long-term impacts of pregnancy, adhere to medical recommendations, maintain social connections, and remain engaged with their families (4–6).

Poor self-care during pregnancy increases the risk of conditions such as anemia, urinary tract infections, hypertension, and chronic energy deficiency (7). Self-care deficits are also linked to obstetric complications such as bacterial vaginosis, pyelonephritis, preeclampsia, oligohydramnios, polyhydramnios, placenta previa, third-trimester hemorrhage, diabetes mellitus, thyroid dysfunction, and maternal heart disease, which may result in preterm birth (5,8). Self-care behavior in pregnancy is defined as compliance with care programs and practices aimed at ensuring the health of both mother and fetus during pregnancy, childbirth, and postpartum (9).

Low self-care can negatively affect maternal emotional health, fetal development, and survival. Kusrini et al. demonstrated that mothers with poor self-care were more likely to give birth to low birth weight (LBW) babies, undergo caesarean delivery, and fail to provide exclusive breastfeeding. Poor self-care behavior is therefore directly related to maternal morbidity and mortality (10).

Globally, maternal mortality remains a major challenge, particularly in low-income countries such as Indonesia. Indonesia ranks third in ASEAN for the highest Maternal Mortality Rate (MMR). In 2020, Indonesia recorded an MMR of 189 per 100,000 live births, significantly higher than Malaysia, Singapore, and Vietnam, which have achieved rates below 100 per 100,000 live births. By 2023, Indonesia had still not reached its reduction target, recording 3,572 maternal deaths (11).

Complications such as hypertension, gestational diabetes, infections, and postpartum hemorrhage remain leading causes of maternal and neonatal morbidity and mortality. While many of these are preventable, a lack of timely monitoring and appropriate intervention persists in low-income countries. A significant challenge is that many women lack adequate information to identify danger signs early or do not know the appropriate actions to take. The ability to access, evaluate, and apply health information for better decision-making is referred to as health literacy (12).

Health literacy is crucial for improving individual and community health outcomes. It is defined as the ability to acquire health knowledge and make informed decisions regarding personal health (10,13,14). For pregnant women, adequate health literacy facilitates recognition of danger signs, adherence to medical advice, and engagement in self-care practices that minimize complications.

In Indonesia, the Human Development Index (HDI)—closely related to literacy—remains relatively low at 74.39 in 2023 (15). Low health literacy is associated with increased emergency visits, longer hospital stays, poorer health outcomes, and limited use of preventive services. It also correlates with reduced antenatal care (ANC) visits during pregnancy (6). Despite its importance, health literacy among Indonesian pregnant women remains under-researched, particularly in maternal health contexts.

Pregnant women with low health literacy are at greater risk of poor self-management, malnutrition, lack of folic acid supplementation, delayed prenatal care, and higher rates of hospitalization (10,16,17). Self-care during pregnancy encompasses physical, psychological, and spiritual dimensions, including hygiene, ANC visits, nutrition, rest and activity, and adherence to prescribed medications and vitamins. The first and most critical determinant of self-care is health literacy (18).

However, national-level data on maternal self-care practices in Indonesia are lacking. In Makassar City, data show increasing cases of LBW and declining tetanus toxoid (TT) immunization coverage, indicating insufficient maternal self-care. Given this situation, studying the quality of self-care behavior among pregnant women is of critical importance.

Improving self-care can be achieved by enhancing health literacy. Literacy skills enable pregnant women to access, understand, and use health information effectively. Health literacy is vital not only for individuals but also for policymakers and healthcare professionals involved in health promotion, disease prevention, and maternal care. Increased health literacy can improve maternal quality of life, reduce healthcare costs, and lower mortality rates (9). Inadequate health literacy often results in poor understanding of antenatal care (ANC), mismanagement of medical conditions, and harmful self-care practices. Conversely, women with good health literacy are more likely to maintain proper nutrition, attend prenatal classes, and comply with ANC schedules.

The Kassi-Kassi Primary Health Care Center in Makassar City serves a large number of pregnant women and records high rates of hypertension and depression risk. Preliminary interviews indicated that maternal health literacy in this area remains low. Although some international studies have explored the relationship between health literacy and self-care in pregnancy, research in Indonesia—particularly in Makassar City—is still limited. Moreover, previous studies have often used general literacy instruments rather than tools specifically designed for pregnant women.

Therefore, this study employs an instrument specifically developed to measure maternal health literacy and examines its relationship with self-care among pregnant women. Based on the above background, this research aims to investigate the relationship between health literacy and self-care in pregnant women.

## Materials and Methods

This study employed quantitative design with a cross-sectional approach, conducted at the Kassi-Kassi Health Center in South Sulawesi from May 10 to June 7, 2024. From a total population of 80 postpartum mothers, 60 participants were selected using purposive sampling.

Inclusion criteria: Currently pregnant, residing in the study area, able to communicate in Bahasa Indonesia, and willing to provide informed consent.

Exclusion criteria: Women with severe medical or psychiatric conditions that could interfere with participation.

Health literacy was considered the independent variable, while self-care behavior served as the dependent variable.

**Instruments**: Data were collected using two questionnaires. The maternal Health Literacy and Pregnancy Outcome Questionnaire (MHLAPQ) developed by Mojoyinola (2011). This tool contains 26 items measuring maternal health literacy, healthy pregnancy, and pregnancy outcomes. Items are rated on a 5-point Likert scale (1 = strongly disagree, 2 = disagree, 3 = don't know, 4 = agree, 5 = strongly agree). Maternal health literacy levels were assessed based on average scores for individual items and overall responses. The MHLAPQ demonstrated a Cronbach's alpha of 0.81 in this study, indicating good reliability. Its use in other countries has consistently shown strong internal consistency: a Persian version reported Cronbach's alpha values of 0.89 for maternal health literacy and 0.67–0.87 for pregnancy outcomes (19), while a Nigerian study reported 0.822 for the maternal health literacy section (20). A Cronbach's alpha above 0.70 is generally considered acceptable, and the value of 0.81 obtained in this study confirms its reliability in this population.

On the other hand, the self-care questionnaire was developed by the researchers. It consisted of 29 items scored on a 4-point Likert scale (1 = strongly disagree, 2 = disagree, 3 = agree, 4 = strongly agree). The validity test yielded r = 0.443, and the Cronbach's alpha was 0.739, indicating acceptable reliability.

**Data analysis**: Data were analyzed using the Spearman correlation test with a significance level of 0.05, processed through SPSS version 26.

The study strictly adhered to the ethical standards for research involving human subjects and complied with the principles of the Helsinki Declaration. Ethical approval was obtained from the Health Research Ethics Committee, Faculty of Medicine and Health Sciences, with approval number C.048/KEPK/FKIK/V/2024. Written informed consent was obtained from all participants before their involvement in the study. All individual information was kept strictly confidential and was not reported in the manuscript.

## Results

Table 1 summarizes the characteristics of pregnant women in this study. The mean (SD) age of participants was 26.67 (4.16) years (range: 18–44 years). Most respondents had completed senior high school (n = 64; 97.0%). The majority were housewives (n = 54; 81.8%). In terms of delivery type, artificial delivery (caesarean section or assisted) was most common (n = 36; 54.5%). Regarding parity, most participants were multiparous (n = 46; 69.7%).

**Table 1:** Distribution of Characteristics of Pregnant Mothers (N= 66)

Characteristics	Frequency (%)
Age	
18-44	66 (100)
Education	
Elementary school	1 (1.5)
Junior High School	1 (1.5)
Senior High School	64 (97.0)
Work	
Staff	5 (7.6)
Self-employed	7 (10.6)
Housewife	54 (81.8)
Types of Childbirth	
Normal	30 (45.5)
Artificial (Tool Assistance)	36 (54.5)
Number of children	
Primipara	20 (20.3)
Multipara	46 (69.7)

Table 2 presents an overview of maternal health literacy during pregnancy based on individual questionnaire items. The analysis shows that participants generally scored an average of 4 (“agree”) in several areas, including basic reading and writing skills, the ability to interpret simple health information, knowledge of vaccinations, baby and postpartum care, and achieving birth weights above 2500 g.

**Table 2:** Distribution Based on Item Answers regarding Health Literacy in Pregnant Women (N=66)

Question Items	Median	Minimum	Maximum
I can read and write.	4.00	1	5
I can do operation mathematics basic (eg. count amount fruits and vegetables or meat based on need during pregnancy).	4.00	4	5
I can understand terms medical base for pregnancy (eg anemia, edema, hypertension, etc.)	3.00	1	5
I can read and understand related materials with health with Good	4.00	2	5
I can understand and interpret information health basic and simple (eg. brushing teeth, washing hands, etc.)	4.00	3	5
I have ability reading, understanding and behaving in accordance with teachings provider service health	2.00	1	5
I can read book health and get skills cleanliness personal and cleanliness food during pregnancy and postpartum labor	3.00	1	5
I have ability read and understand sign danger during pregnancy (eg anemia, pale congested breath, pressure blood height, swelling, bleeding and labor premature, etc.)	2.00	1	5
I have ability for reading, understanding and interpreting Recipe and instructions medical	4.00	3	5
I have ability read and understand date visit I next (for example for vaccination, services health, check-up health, etc.)	4.00	1	5
I have Enough information knowledge about pattern food needed during pregnant and after pregnant	2.00	1	5
I have sufficient skills for prepare a proper diet and use it	2.50	1	5
I have sufficient knowledge and skills about method nurse baby after born (eg. breastfeeding, bathing, etc.)	4.00	3	5
I have ability for look for information health from book or internet	3.00	1	5
If I have sign danger pregnancy, me visit health center or doctor private	4.00	3	5
I have Enough information sign danger moment pregnant	3.00	1	5
I attended prenatal classes in regular	2.00	1	5
I have Enough information about vaccination during pregnancy	4.00	2	5
I can take action for overcome one sign danger during pregnancy	3.00	1	5
I have ability for detect sign danger during pregnancy	2.00	1	5
I have got proper prenatal care time	3.00	1	5
I do not experience born dead during pregnancy	3.00	2	5
I gave birth appropriate time	4.00	2	5
I had a safe and comfortable delivery process.	4.00	4	5
I have Lots Of problem during pregnancy	4.00	2	4
I gave birth baby with normal body weight (>2500 g)	4.00	1	5

In contrast, lower levels of health literacy were observed in areas with an average score of 2 (“disagree”), particularly in interpreting more complex information provided by healthcare professionals, recognizing danger signs and symptoms of pregnancy, understanding appropriate nutritional needs, managing dietary patterns, and engaging in pregnancy exercise.

Table 3 provides a detailed overview of respondents' self-care behaviors during pregnancy. Several practices were reported with relatively high frequency, reflected in a median score of 3.00, including regularly seeking pregnancy-related information from doctors or midwives, reporting general pregnancy symptoms to healthcare providers, performing routine breast self-examinations, maintaining skin hygiene, trimming nails weekly, taking daytime rest, and adhering to prescribed medications or iron supplements.

**Table 3:** Distribution Based on Item Answers Regarding Self Care in Pregnant Women (N=66)

Question Items	Median	Minimum	Maximum
Antenatal Care (ANC) Visit			
Mother discusses complaint pregnancy to doctor / midwife / nurse	2.00	1	4
Mother always make promise with doctor / midwife in charge Mother	2.00	1	4
Mother asked information around pregnancy to doctor / midwife	3.00	2	4
Mother reported common symptoms felt related pregnancy to doctor / nurse / midwife	3.00	1	4
**Personal Hygiene**			
Mother combs her hair at least 2 times a day	2.00	2	4
Mother washes her hair at least twice a week using shampoo	3.00	2	4
Mother dries her hair and scalp after washing her hair	1.00	1	4
Mother brushes her teeth after eating and drinking, and before sleeping	2.00	1	4
Mother takes a bath at least twice a day	2.00	2	4
When mother taking a bath, cleaning areas folds skin (eg. armpit, bottom breasts, and area genitalia)	3.00	2	4
Mother cleans up after urinating and defecating with clean water	3.00	2	4
Mother uses trousers in easy absorb sweat	2.00	1	4
Mother changed trousers in when feel moist	3.00	2	4
Mother cleans area genitals with soap	3.00	1	4
Mother cleans breast a week very	3.00	2	4
Mother checks breast in a way regular	3.00	1	4
Mom changed her bra more from 2 times in a day	2.00	1	4
Mom wears a bra that doesn't fit too strict	3.00	2	4
Mom cuts her nails once a week once	3.00	2	4
Mother soaks her feet in warm water twice a week	1.00	1	4
Mother massages foot area a week very	2.00	1	4
**Nutrition**			
Mothers drink at least 2 glasses of water every day. day	4.00	2	4
Drink 1 glass of milk a day	2.00	1	4
Drink alcohol	4.00	4	4
Eating fruits and vegetables 5 bowls of vegetables a day	2.00	1	4
Eat more Lots meat without fat or egg	2.00	1	4
Activity Physique			
Exercise 30 minutes 3x/week	1.50	1	4
Mother rests during the day day	3.00	3	4
Stress Management			
Mother ever experiencing stress	3.50	3	4
When mother is overly stressed whether influence fetus	3.50	3	4
Health Responsibility			
Eating drug or pill substance iron as suggested by power health	3.00	1	4
Smoking (cigarettes) or local cigarettes)	4.00	4	4
Mother did inspection health in the first trimester	2.00	2	4
Mother did inspection health teeth at the beginning pregnancy	2.00	1	4

In contrast, other self-care behaviors showed lower levels of practice, with a median score of 2.00. These included discussing pregnancy-related complaints with healthcare providers, scheduling regular appointments with midwives or doctors, soaking feet in warm water, and consuming key nutritional sources such as milk, fruits, and meat. Pregnant women were also less likely to engage in regular physical exercise or undergo early dental check-ups.

Spearman correlation test results showed health literacy had strong correlation with self-care behavior, with an r-value of 0.849 (P<0.001).

## Discussion

Health literacy is strongly associated with self-care behaviors among pregnant women, with higher levels of health literacy linked to better self-care practices (21,22). This ability plays a crucial role in improving maternal health outcomes. Women with higher health literacy demonstrate greater knowledge of appropriate self-care during pregnancy, such as maintaining a balanced diet, engaging in physical activity, and attending regular health check-ups (21,23). Previous studies also reported a positive correlation between health literacy, knowledge, and self-care, indicating that women with better health literacy are more aware of essential practices and risk avoidance compared to those with lower literacy levels (22,23).

Moreover, health literacy significantly influences adherence to medical advice during pregnancy, including compliance with prenatal vitamin intake and attendance at routine antenatal appointments (13). Women with lower health literacy often experience difficulties in following medical recommendations, which increases the risk of adverse maternal outcomes. This highlights the importance of providing accessible health information to ensure consistent self-care practices. Additionally, maternal health literacy has been positively associated with adherence to recommended antenatal care visits (24).

This study also identified specific gaps in maternal health literacy, particularly in critical literacy. Many pregnant women struggled to fully understand or apply information regarding nutritional needs, pregnancy danger signs, and participation in prenatal classes. Inadequate critical health literacy poses significant risks during pregnancy and the postpartum period if not addressed through appropriate interventions. The lack of critical health literacy may be related to limited exposure to critical thinking practices, education patterns, and family upbringing, all of which strongly influence cognitive development. These findings are consistent with previous research showing that communicative and critical health literacy are associated with improved self-care management in pregnant women (10).

Other studies further support these findings, indicating that health literacy significantly influences self-care behavior, with individuals possessing higher literacy levels more likely to practice effective self-care (10,16,21). Importantly, health literacy can be improved through education tailored to individual needs. Such interventions should consider educational level, cultural background, gender, occupation, and age. Nurses, in particular, are encouraged to employ teaching methods that are simple, diverse, and customized to these factors to optimize maternal self-care outcomes. This study concludes that higher maternal health literacy is strongly associated with improved self-care behavior during pregnancy, underscoring the importance of enhancing health literacy to promote better maternal health outcomes in low-income settings.
